# Prevention of Fascial Retraction in the Open Abdomen with a Novel Device

**DOI:** 10.1155/2020/8254804

**Published:** 2020-10-21

**Authors:** Anita Hees, Frank Willeke

**Affiliations:** Department of General, Visceral and Vascular Surgery, Marien Hospital, 57072 Siegen, Germany

## Abstract

The open abdomen requires intensive and specific treatment efforts. Long hospital admissions, treatment duration, high mortality rates, deferred and delayed wound closures with alloplastic materials or elaborate closure techniques, and the need for subsequent surgical procedures justify and call for implementation of new therapy options. The case presented here demonstrates the use of a new product (Fasciotens Abdomen) to prevent fascial retraction in the open abdomen of an extubated, conscious patient with four-quadrant peritonitis after perforated appendicitis. Controlled, anteriorly directed fascial traction of 50-60 Newtons prevented fascial retraction during open treatment of the abdomen. Once edema was reduced, abdominal closure was completed without difficulty. This new form of therapy was well tolerated by the patient and led to a markedly more rapid abdominal closure without mesh or abdominal wall reconstruction.

## 1. Introduction

Treating the open abdomen is challenging and requires all means available for rapid abdominal closure [1]. To date, negative pressure wound therapy has been well accepted and commonly used [2, 3]. Delayed closure often requires alloplastic material or can be impeded, and ventral hernias may result [4, 5]. A prolonged open abdomen results in bowel adhesions, enteroatmospheric fistulas, and/or loss of domain [6–12]. Mortality ranges from 12 to 40%; septic origin is associated with higher mortality [1]. Early closure of the open abdomen has yielded good results in trauma patients. Fewer complications and lower mortality rates were observed [13–15]. A novel device called Fasciotens Abdomen was developed to prevent fascial retraction in the open abdomen. In vivo animal testing achieved positive results. Eickhoff et al. found beneficial effects regarding closing force and abdominal circumference in a patient cohort treated with Fasciotens versus controls. Changes in ventilation or vital parameters and/or histological tissue damage were not observed. Eickhoff et al. concluded that Fasciotens would enable higher rates of primary closure as well as earlier closure [16].

The case presented here details the application of Fasciotens Abdomen in a conscious patient with an open abdomen including feasibility and effects of use.

## 2. Case Presentation

**Figure 1:**
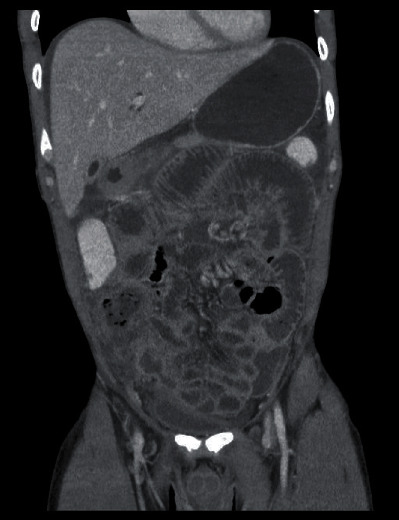
CT abdomen with evidence of ileus most localized in the area of the ileocecal valve with questionable characteristics. No evidence of intra-abdominal free air or peri/subhepatic ascites.

A 36-year-old male presented to the internal medicine section of the Emergency Department with nonspecific abdominal complaints. There was no previous medical history. Abdominal examination revealed diffuse abdominal pain. Laboratory examination showed increased CRP of 15.2 mg/dL with normal WBC count of 6,570/*μ*l. The patient was admitted for observation. Follow-up laboratory studies the next day showed clear increases in inflammatory parameters with WBCs of 16,900/*μ*l and CRP of 43.61 mg/dl. Nonspecific abdominal symptoms continued. The patient developed an acute abdomen overnight and was evaluated surgically. CT abdomen (Figure 1) showed ileus near the ileocecal valve with questionable characteristics. There was no intra-abdominal free air or peri/subhepatic ascites.

Because of the imaging and increasing symptoms, an exploratory laparotomy was performed on the third day of treatment. We decided to a primarily open abdomen because of the pronounced ileus in combination with the acute abdomen, the expected disease severity, and the small amount of space expected in the abdomen. Immediately preoperatively, the patient received antibiotics with Cefuroxim 1.5 g and Clont 500 mg intravenous. The antibiotic therapy was continued: Cefuroxim 1.5 g 3 times a day and Clont 500 mg 2 times a day. A transverse upper abdominal laparotomy (with the upper abdominal transverse laparotomy, we have had very good experiences with regard to the abdominal fascia dehiscence and the development of scar hernias) diagnosed a fecal 4-quadrant peritonitis with cloudy liquid and perforated gangrenous appendicitis. There was also severe inflammation in the cecal region, terminal ileum, and the colon ascendens (Björck 2 c) [17]. So, we decided an open right hemicolectomy followed with mechanical reinforced terminolateral ileotransverse anastomosis. In addition, extensive lavage was performed and laparostomy was placed with Vicryl mesh (Ethicon, Cincinnati, Ohio, USA) implantation. Due to pronounced edematous swelling of the abdomen, it was left open. There was no option for fascial closure with an expected compartment and also the necessary recurrent lavage with a 4-quadrant peritonitis. The initial fascial distance measured 12 cm under full relaxation. Planned surgical lavage was performed on the following day for fecal peritonitis. The first abdominal lavage at the following day showed that there was still 4-quadrant peritonitis and also pronounced edema of the intestine. Closing the abdomen was not possible or considered. Some more lavages are necessary.

**Figure 2:**
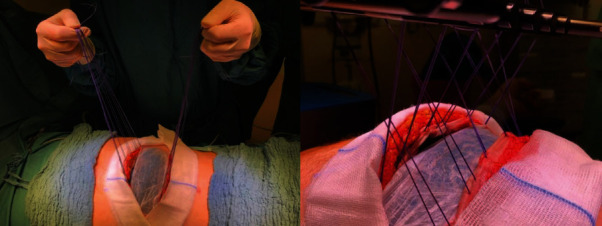
Intraoperative application of Fasciotens Abdomen. Surgical suture (Vicryl USP 1 or 2) was attached to the mesh. The suture was connected in turn to the system and a tensile force of 50-60 Newtons was applied to the abdominal wall.

**Figure 3:**
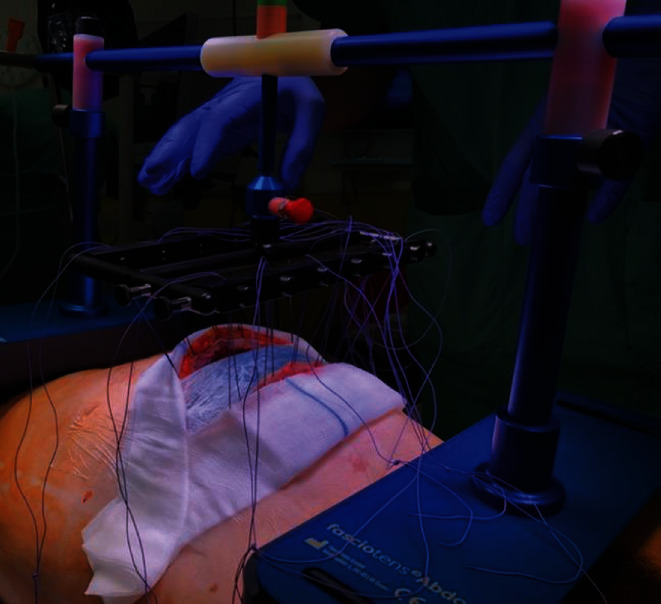
Application of Fasciotens Abdomen.

Because there are potential issues with care on-demand relaparotomy concerning abdominal wall and fascial retraction and the condition of the abdominal wall, we decided to use the Fasiotens Abdomen. During this procedure, the Fasciotens Abdomen (Fasciotens GmbH, Essen, Germany) was applied to prevent abdominal wall retraction during open treatment (Figures 2 and 3).

**Figure 4:**
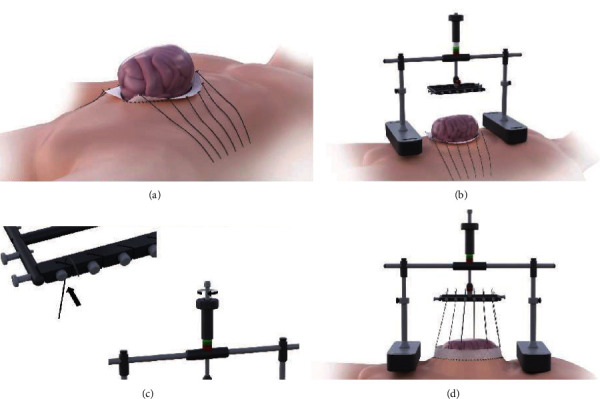
First, a surgical mesh stripe is attached to each of the fascial edges. Sutures USP 1 and 2 are attached to the mesh for traction (a). Fasciotens Abdomen is assembled and placed on chest and anterior pelvic ring (b). Sutures are clamped in, and traction is adjusted with a screw mechanism (c). The applied overall traction is displayed on a scale and constantly controlled and adjustable (d). Image supplied by Fasciotens (all rights reserved).

For intraoperative application of the system, the primary interposed mesh applied to the posterior fascia was split, flipped over, and also affixed to the anterior fascial layer. Six sutures (Vicryl USP 1 or 2, Ethicon, USA) were applied to both sides of the mesh and connected to the Fasciotens Abdomen. Suprasorb CNP drainage film (Lohmann&Rauscher GmbH & Co. KG, Neuwied, Germany), wet gauze and sterile adhesive film were used as entire visceral protection layer and to cover the wound. The product was aligned according to the manufacturer's recommendations based on defect localization and patient size. A tensile force of 50-60 Newtons was set and applied to the abdominal wall. There were no technical difficulties during application.  Figure 4 illustrates the installation of the device.

Postoperative therapy was performed in the Intermediate Care Station for extubated and conscious patients. During the daytime, treatment periods were three hours long with interim respite periods of one hour each, in which the stand was uncoupled from the suture holder on the emergency release button. At night, therapy was also interrupted. The sutures were brought diagonally and fixed under tension to enable further, albeit reduced tension during the night. Regional anesthesia was administered via a thoracic peridural catheter. The antibiotics intravenously with Cefuroxim and Clont are passed on, and he also received intensive physiotherapy treatment. The tensile force setting was continuously monitored along the color scale and adjusted as needed.

**Figure 5:**
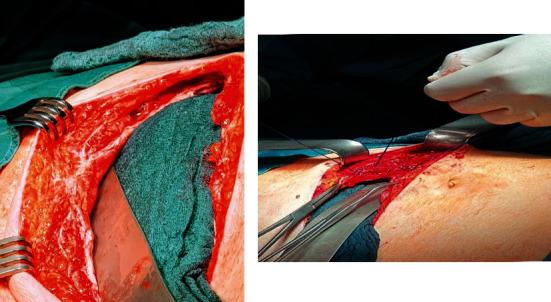
Abdominal wall closure after 6 days. Note the differentiation of the anterior and posterior fascial layers.

During the remaining course, three more lavages were carried out over six days, with declining intra-abdominal edema. During the final lavage on the sixth day of the Fasciotens Abdomen treatment, the wound and fascial margins were inspected along with the abdomen. Intraoperative measurement of the fascial distance under complete relaxation was 4 cm, and abdominal wall closure was completed (Figure 5) There was no macroscopic evidence of fascial necrosis or damage. The anterior and posterior fascial layers were clearly identifiable and could thus be sequentially closed in two layers.

During therapy, there were no difficulties in use by either staff or patient. At times, decoupling for therapeutic breaks was carried out by the patient himself. The patient was discharged after another 8 days. Outpatient follow-up showed a normal wound and a symptom-free patient. He has recovered very well and has no consequences regarding the serious illness.

## 3. Discussion

The case presented here demonstrates the effective treatment of an open abdomen with a newly developed product that prevents fascial retraction by applying anteriorly directed traction while the abdomen is left open. The traction vector enables immediate fascial traction with simultaneous intra-abdominal volume enlargement and pressure relief during the laparostoma phase.

The main difficulties of an open abdomen are the increased complication rate with prolonged duration and the cumulative retraction of the abdominal wall [6–12]. Because of prolapsing organs, medially directed traction is not possible without increasing intra-abdominal pressure. The typical duration of treatment can be estimated based on the relevant literature. Verdam et al. reported on 18 patients with peritonitis after bowel perforation. The average duration of the open abdomen there was 10 days (range 2-39 days). Using an abdominal reapproximation system (ABRA, Canica Design, Almonte, Ontario, Canada) in combination with a VAC abdominal dressing (Kinetic Concepts, Inc. San Antonio, TX) or Bogota bag, average closure was attained after a further 15 days (range 7-30 days) [18]. In a group of 157 patients with abdominal aortic aneurysm, Acosta et al. reported a median two days of open abdomen and nine subsequent days till abdominal wall closure (IQR 6-15) [19]. In the case presented here, in contrast, closure was completed immediately after the need for lavages resolved. Mesh-mediated traction or any other system to recover the abdominal wall was not required. Over the course of revisions, there was even a reduction in laparostomal width. Although this measurement is susceptible to error due to intra-abdominal volume, it must be emphasized nevertheless that the typical widening of the fascial margins was actually reversed, and instead, approximation occurred. Mesh interposition or plastic reconstruction was unnecessary with the successful abdominal wall closure. Closure was carried out on the day of the final lavage.

We have little experience with the Wittmann Patch and none with ABRA device. We only used the Wittmann Patch a few times and discarded it as not very successful: (1) The patch was torn out of the fascia and (2) it causes a horizontal pulling effect, which in turn increases the intra-abdominal pressure.

In the other reported cohorts, there were further delays and revisions required once intra-abdominal findings are normalized. The number of further revisions needed was given only by Acosta et al., who reported a median of 4 revisions prior to abdominal wall closure (IQR 2-6) [19].

From an economical point of view of the DRG (German diagnostic case system) billing system, duration of admission is also relevant. The average length of stay was 65 days for Verdam et al., with 20 days in the intensive care unit [18]. Acosta et al. reported median stays of 31 and 15 days, respectively [19]. The case presented here had a total stay of 16 days, including 6 days in the intermediate care station. This reduction in stay is already considerable when compared to the relevant literature. However, the young patient presented here had no comorbidity and did not require ventilation, thus falls into a lower DRG rating. From an economic perspective, the presented case seems promising. However, larger trials are needed to analyze cost-effectiveness.

One additional innovation in the presented case involves the placement of the medical device on the thorax and anterior pelvic ring. According to the manufacturer, this is the first application in a conscious patient. In the absence of comparable products and without mechanical ventilation parameters, only subjective sensations can be assessed in spontaneously breathing, conscious patients. This patient tolerated the treatment well, including the thoracic and pelvic pressure. He did not require analgesia beyond that given through the peridural catheter. There was no apparent extracare requirement for nursing or medical care in the intermediate care unit. Disassembly of the system for the patient's desired treatment breaks was often carried out by the patient himself. Handling of the product can also be described as predominantly self-explanatory.

Negative pressure wound therapy (NPWT) is frequently used in open abdomen patients but not the favored treatment option in our center. We have had some bad experiences with it, for example, intestinal perforation. Fasciotens representatives reported that several centers have combined NPWT successfully with Fasciotens Abdomen. However, there is no data about combining both treatments in the pertinent literature yet. Future studies should focus on this and evaluate possible beneficial effects.

Limitations include the fact that this is an individual case. The absence of complicating factors or comorbidity should also be considered. Longer follow-up and prospective and comparative studies with other treatment options are not available. According to the manufacturer, however, some are already underway. Rate of closure, duration of treatment, effects of traction on the fascia, and possible incisional hernias during the course of treatment should be particularly noted in larger patient cohorts.

It should be pointed out again that early and direct abdominal wall closure is the fundamental medical benefit for young patients. The basic principle for preventing fascial retraction thus promises substantial improvements in treatment of the open abdomen.
